# Antifungal Potential of Copper(II), Manganese(II) and Silver(I) 1,10-Phenanthroline Chelates Against Multidrug-Resistant Fungal Species Forming the *Candida haemulonii* Complex: Impact on the Planktonic and Biofilm Lifestyles

**DOI:** 10.3389/fmicb.2017.01257

**Published:** 2017-07-11

**Authors:** Rafael M. Gandra, Pauraic Mc Carron, Mariana F. Fernandes, Lívia S. Ramos, Thaís P. Mello, Ana Carolina Aor, Marta H. Branquinha, Malachy McCann, Michael Devereux, André L. S. Santos

**Affiliations:** ^1^Laboratório de Investigação de Peptidases, Departamento de Microbiologia Geral, Instituto de Microbiologia Paulo de Góes, Universidade Federal do Rio de Janeiro Rio de Janeiro, Brazil; ^2^Programa de Pós-Graduação em Bioquímica, Instituto de Química, Universidade Federal do Rio de Janeiro Rio de Janeiro, Brazil; ^3^The Inorganic Pharmaceutical and Biomimetic Research Centre, Focas Research Institute, Dublin Institute of Technology Dublin, Ireland; ^4^Chemistry Department, Maynooth University, National University of Ireland Maynooth, Ireland

**Keywords:** *Candida haemulonii* complex, metal-based drugs, 1,10-phenanthroline, antifungal activity, anti-virulence, biofilm

## Abstract

*Candida haemulonii, Candida haemulonii* var. *vulnera* and *Candida duobushaemulonii*, which form the *C. haemulonii* complex, are emerging etiologic agents of fungal infections known to be resistant to the most commonly used antifungals. The well-established anti-*Candida* potential of metal complexes containing 1,10-phenanthroline (phen) ligands encouraged us to evaluate different copper(II), manganese(II), and silver(I) phen chelates for their ability to inhibit planktonic growth and biofilm of *C. haemulonii* species complex. Two novel coordination complexes, {[Cu(3,6,9-tdda)(phen)_2_].3H_2_O.EtOH}_n_ and [Ag_2_(3,6,9-tdda)(phen)_4_].EtOH (3,6,9-tddaH_2_ = 3,6,9-trioxaundecanedioic acid), were synthesized in a similar fashion to the other, previously documented, sixteen copper(II), manganese(II), and silver(I) chelates employed herein. Three isolates of each *C. haemulonii* species complex were used and the effect of the metal chelates on viability was determined utilizing the CLSI standard protocol and on biofilm-growing cells using the XTT assay. Cytotoxicity of the chelates was evaluated by the MTT assay, employing lung epithelial cells. The majority of the metal chelates were capable of interfering with the viability of planktonic-growing cells of all the fungal isolates. The silver complexes were the most effective drugs (overall geometric mean of the minimum inhibitory concentration (GM-MIC) ranged from 0.26 to 2.16 μM), followed by the manganese (overall GM-MIC ranged from 0.87 to 10.71 μM) and copper (overall GM-MIC ranged from 3.37 to >72 μM) chelates. The manganese chelates (CC_50_ values ranged from 234.51 to >512 μM) were the least toxic to the mammalian cells, followed by the silver (CC_50_ values ranged from 2.07 to 13.63 μM) and copper (CC_50_ values ranged from 0.53 to 3.86 μM) compounds. When tested against mature biofilms, the chelates were less active, with MICs ranging from 2- to 33-fold higher levels when compared to the planktonic MIC counterparts. Importantly, manganese(II), copper(II), and silver(I) phen chelates are relatively cheap and easy to synthesize and they offer significant antifungal chemotherapeutic potential for the treatment of highly resistant pathogens.

## Introduction

The global incidences of invasive candidiasis has increased considerably in recent decades, being the fourth and sixth leading cause of nosocomial blood infections in the United States of America and Europe, respectively (Caggiano et al., 2015). New advances in medicine have improved the survival rates of innumerous patients, but also resulted in an augment in the number of immunocompromised individuals, who are extremely susceptible to systemic mycoses (Richardson and Lass-Flörl, 2008; Sanguinetti et al., 2015). Infections caused by non-*albicans Candida* species, such as *Candida parapsilosis, Candida glabrata, Candida tropicalis*, and *Candida krusei*, are becoming increasingly more common in hospital settings (Abu-Elteen and Hamad, 2012; Ramos et al., 2015). This new scenario constitutes a clinical challenge, since such non-*albicans Candida* species are more resistant to the different classes of antifungal drugs that are currently available (Abu-Elteen and Hamad, 2012; Ramos et al., 2015). This trend is also being observed for fungal infections caused by other uncommon species such as those belonging to the *Candida haemulonii* complex (*C. haemulonii, Candida duobushaemulonii* and *Candida haemulonii* var. *vulnera*). The risk or predisposing factors associated with fungemia caused by *C. haemulonii* complex include mechanic ventilation, serious conditions such as cancer and immunodeficiency and the use of central venous catheters (Almeida et al., 2012; Cendejas-Bueno et al., 2012; Muro et al., 2012). Given the epidemiological profile of the *C. haemulonii* complex and the fact that it is resistant to such a broad spectrum of the state-of-the-art antifungals (e.g., amphotericin B, flucytosine, fluconazole, itraconazole, voriconazole, micafungin, and caspofungin) (Giusiano et al., 2005; Khan et al., 2007; Kim et al., 2009; Ruan et al., 2010), it is imperative that the scientific community examine alternative therapeutic paths for it treatment. Aggravating this scenario, recently, our research group described the ability of *C. haemulonii* species complex to form biofilm on inert substrate (Ramos et al., 2017). From a medical viewpoint, the microbial biofilm architecture is a complex and robust structure, which is extremely resistant to different classes of drugs (e.g., antifungals and disinfectants), host immune attack (e.g., antibodies, antimicrobial peptides, and complement proteins) and several hostile environmental stressors (e.g., dehydration and radiation; Nett, 2014; Ramage et al., 2014; Mello et al., 2017). Anti-biofilm strategies able to prevent and/or eradicate fungal biofilms in medical devices are urgently required (Ramage et al., 2014; Ramos et al., 2017).

The anti-*Candida* activity of transition metal chelates containing 1,10-phenanthroline (phen) ligands is already well-established, and it is known that copper(II)-, manganese(II)-, and silver(I)-phen chelates are potent growth inhibitors of *C. albicans, C. glabrata, C. tropicalis*, and *C. krusei* (Geraghty et al., 2000; McCann et al., 2000, 2012; Coyle et al., 2003). These compounds affected mitochondrial function, reduced cytochrome *b* and *c* synthesis, induced respiratory uncoupling and increased cell wall permeability in the fungal cells (McCann et al., 2004; Creaven et al., 2007). Given the proven anti-*Candida* profile of phen-based drugs, we decided to examine the anti-*C. haemulonii* species complex therapeutic potential of a series of copper(II), manganese(II), and silver(I) phen chelates on both planktonic growth and biofilm of nine Brazilian clinical isolates of the *C. haemulonii* complex. The hypothesis of our study is based on the premise that clinical isolates belonging to the *C. haemulonii* complex often exhibit resistance to different classes of antifungal agents commonly used in medical practice and, therefore, there is an urgent need to find new active compounds against these emerging fungal species.

## Materials and methods

### Chemistry

Chemicals were purchased from commercial sources and used as received without further purification. Infrared (IR) spectra were recorded in the region 4,000–370 cm^−1^ on a Perkin Elmer System 2000 FT spectrometer and using anhydrous potassium bromide to form solid KBr discs. Elemental analysis (CHN) was carried out for all the complexes using a FLASH EA 1112 Series Elemental Analyser with Eager 300 operating software. Room temperature, solid state magnetic susceptibility measurements for the Cu(II) compounds, [Cu(3,6,9-tdda)].H_2_O and {[Cu(3,6,9-tdda)(phen)_2_].3H_2_O.EtOH}_n_ (**7**), were made using a Johnson Matthey Magnetic Susceptibility Balance.

The following metal chelates were prepared using previously published methods: [Cu(ph)(phen)(H_2_O)_2_] (**1**) (phH_2_ = phthalic acid) (Kellett et al., 2012); [Cu(ph)(phen)_2_].3H_2_O.2EtOH (**2**) (Kellett et al., 2011); [Cu(isoph)(phen)_2_].6H_2_O.EtOH (**3**) (isophH_2_ = isophthalic acid) (Kellett et al., 2011); [{Cu(phen)_2_}_2_(terph)](terph).13.5H_2_O.2EtOH (**4**) (terphH_2_ = terephthalic acid) (Kellett et al., 2011); [Cu_2_(oda)(phen)_4_](ClO_4_)_2_.2.76H_2_O.EtOH (**5**) (odaH_2_ = octanedioic acid) (Devereux et al., 1999); [Cu(phendione)_3_](ClO_4_)_2_.4H_2_O (**6**) (phendione = 1,10-phenanthroline-5,6-dione) (McCann et al., 2004); [Mn(ph)(phen)(H_2_O)_2_] (**8**) (Devereux et al., 2000); [Mn(ph)(phen)_2_(H_2_O)].4H_2_O (**9**) (Devereux et al., 1999); [Mn_2_(isoph)_2_(phen)_3_].4H_2_O (**10**) (Devereux et al., 2000); {[Mn(phen)_2_(H_2_O)_2_]}_2_(isoph)_2_(phen).12H_2_O (**11**) (Devereux et al., 2000); [Mn(tereph)(phen)_2_].5H_2_O (**12**) (Leon, 2000); [Mn_2_(oda)(phen)_4_(H_2_O)_2_][Mn_2_(oda)(phen)_4_(oda)_2_].4H_2_O (**13**) (Casey et al., 1994); {[Mn(3,6,9-tdda)(phen)_2_].3H_2_O.EtOH}_n_ (**14**) (3,6,9-tddaH_2_ = 3,6,9-trioxaundecanedioic acid) (McCann et al., 1997); [Ag(phendione)_2_]ClO_4_ (**15**) (McCann et al., 2004); [Ag(phen)_2_]ClO_4_ (**17**) (McCann et al., 2004) and [Ag_2_(phen)_3_(udda)].3H_2_O (**18**) (uddaH_2_ = undecanedioic acid) (Thornton et al., 2016). The novel complexes, {[Cu(3,6,9-tdda)(phen)_2_].3H_2_O.EtOH}_n_ (**7**) and [Ag_2_(3,6,9-tdda)(phen)_4_].EtOH (**16**), were synthesized using the two-step procedures outlined below. The preparation of all silver compounds was conducted in the absence of light and the products were stored in the dark.

**{[Cu(3,6,9-tdda)(phen)**_2_**].3H**_2_**O.EtOH}**_*n*_ **(7)**. Step (i): Copper(II) acetate hydrate (1.50 g; 7.51 mmol) was dissolved in ethanol (50 mL) and the solution was added to an ethanolic solution (25 mL) of 3,6,9-trioxaundecanedioic acid (3,6,9-tddaH_2_) (2.84 g; 8.95 mmol). The light-green suspension was refluxed for 1 h, and after cooling to room temperature the light-green solid, [Cu(3,6,9-tdda)].H_2_O, was filtered off, washed with cold ethanol and air dried. Yield: 1.88 g (82.97%). % Calculated: C: 31.84, H: 4.68. % Found: C: 32.20, H: 4.60. IR (KBr) ν_max_: 3280, 2930, 1585, 1430, 1330, 1250, 1130, 1090, 1055, 965, 920, 840, 720, 475 cm^−1^. μ_eff_: 1.93 B.M. Solubility: soluble in H_2_O, MeOH, EtOH and insoluble in CHCl_3_, ethyl acetate and acetone. Step (ii): [Cu(3,6,9-tdda)].H_2_O (1.00 g; 3.31 mmol) and phen (2.39 g; 13.26 mmol) were dissolved together in ethanol (50 mL) and the resulting dark-green mixture was refluxed for 2 h. The suspension was cooled and the green solid, {[Cu(3,6,9-tdda)(phen)_2_]3H_2_O.EtOH}_n_(**7**), was filtered off, washed with cold ethanol and air dried. Yield: 1.63 g (66.09%). % Calculated: C: 54.87, H: 5.42, N: 7.53. % Found: C: 54.65, H: 5.63, N: 7.39. IR (KBr) ν_max_: 3982, 3415, 3041, 2901, 1989, 1752, 1618, 1587, 1513, 1421, 1320, 1252, 1221, 1121, 1090, 1077, 1011, 934, 893, 847, 770, 720, 704, 643, 619, 603, 573, 555, 507, 426 cm^−1^. μ_eff_: 1.92 B.M. Solubility: soluble in H_2_O, MeOH, EtOH and insoluble in ethyl acetate and acetone.

**[Ag**_2_**(3,6,9-tdda)(phen)**_4_**].EtOH (16)**. Step (i): Silver(I) acetate (3.00 g; 17.97 mmol) was dissolved in ethanol (30 mL) and the solution was added to an ethanolic solution (25 mL) of 3,6,9-trioxaundecanedioic acid (3,6,9-tddaH_2_) (2.84 g; 8.95 mmol) and the orange-brown suspension refluxed for 3 h. After cooling to room temperature, the light orange-brown powder, [Ag_2_(3,6,9-tdda].2H_2_O, was filtered off, washed with cold ethanol and then air dried. Yield: 3.79 g (44.69%). % Calculated: C: 20.36, H: 2.56. % Found: C: 20.34, H: 2.51. IR (KBr) ν_max_: 3425, 2896, 1614, 1406, 1116 cm^−1^. ^1^H NMR: (D_2_O) δ = 3.81 (s, 4H), 3.56 (s, 8H). Solubility: soluble in hot H_2_O and hot DMSO. Step (ii): [Ag_2_(3,6,9-tdda].2H_2_O (0.50 g; 1.06 mmol) and phen (0.827 g; 4.589 mmol) were dissolved together in ethanol (40 mL) and the mixture refluxed overnight. After cooling to room temperature the mixture was placed in an ice bath. Green [Ag_2_(3,6,9-tdda)(phen)_4_].EtOH (**16**) precipitated and was filtered off, washed with cold ethanol and air dried. Yield: 0.83 g (65.10%). % Calculated: C: 57.92, H: 4.19, N: 9.32. % Found: C: 57.75, H: 5.16, N: 9.02. IR (KBr) ν_max_: 3380, 3046, 2905, 1978, 1804, 1618, 1585, 1509, 1421, 1322, 1263, 1215, 1121, 1077, 1018, 932, 890, 838, 726, 621, 465, 413 cm^−1^. ^1^H NMR (500 MHz, DMSO-d6, 313 K, TMS) δ = 9.12(8H, s), 8.63 (8H, d), 8.08 (8H, s), 7.91 (8H, dd), 3.74 (4H, s), 3.57 (8H, dd). ^13^C NMR (125 MHz, DMSO-d6, 313 K, TMS) δ = 173.41, 151.28, 143.57, 138.03, 129.24, 127.37, 124.74, 71.44, 70.42, 69.74. Solubility: soluble in H_2_O, MeOH, EtOH and insoluble in ethyl acetate and acetone.

### Epithelial cell lineage and microorganisms' growth conditions

Three clinical isolates of each species that form the *C. haemulonii* complex were used in this study: *C. haemulonii* (LIP*Ch*4 from finger nail, LIP*Ch*7 from toe nail, and LIP*Ch*12 from blood), *C. duobushaemulonii* (LIP*Ch*1 from finger nail, LIP*Ch*6 from toe nail, and LIP*Ch*8 from blood) and *C. haemulonii* var. *vulnera* (LIP*Ch*5 from toe nail, LIP*Ch*9 from urine, and LIP*Ch*11 from blood) (Ramos et al., 2015). The fungal isolates were identified by the automatized system VITEK® 2 and then characterized by *ITS1-5.8S-ITS2* gene sequencing (Ramos et al., 2015). Fungal cells were cultured in Sabouraud-dextrose medium under constant agitation (130 rpm) for 48 h at 37°C. Human lung adenocarcinoma A549 cells were maintained in culture flasks containing DMEM medium supplemented with 10% fetal bovine serum at 37°C in an atmosphere of 5% CO_2_.

### Effects of test compounds on planktonic fungal growth

Susceptibility profile assays were performed in accordance with the broth microdilution protocol described in the document M27-A3 published by the Clinical and Laboratory Standards Institutes (CLSI, 2008). Samples of the test chelates were dissolved in DMSO (500 μL) and then serially diluted in a 96-well plate containing RPMI 1640 (Sigma-Aldrich, USA) to give the concentration range of 0.0625-32 mg/L. Aqueous solutions of the metal-free phen ligand and the simple metal salts, MnCl_2_, CuCl_2_, and AgNO_3_, were also screened over the same concentration range. Caspofungin (Sigma-Aldrich) was used as a classical antifungal agent able to block the growth of all clinical isolates of *C. haemulonii* species as previously reported by our research group (Ramos et al., 2015). Water dilutions containing DMSO concentrations corresponding to those used to prepare the drug solutions were assessed in parallel. Untreated and compound-treated fungal cells were incubated at 37°C for 48 h and the minimal inhibitory concentration (MIC) was established as the lowest compound concentration where no cellular growth could be detected by visual inspection in accordance to the CLSI recommendation. The geometric mean of the MIC values (GM-MIC) of each compound against all the fungal isolates was calculated using the software, Microsoft Office Excel 2013.

### Mammalian cell toxicity assay and determination of selectivity indexes

The cytotoxicity was evaluated using the MTT [3-(4,5-dimethylthiazol-2-yl)-2,5-diphenyl tetrazolium bromide] (Sigma-Aldrich) assay (Mosmann, 1983). A549 cells (10^4^) were seeded into tissue culture plates (TPP, Switzerland) and cultured for 24 h at 37°C in a 5% CO_2_ in order to obtain cellular confluence. The wells were then washed twice with DMEM to remove non-adherent cells and the test compounds were added (in concentrations ranging from 0.0313 to 512 μg/mL) to plates containing DMEM, followed by a 48 h incubation period under the same conditions mentioned above. The cellular viability was evaluated by adding MTT to each well and incubating the plates in the dark for 3 h, allowing the viable cells containing active mitochondrial dehydrogenase enzymes to metabolize MTT-tetrazolium salt into purple formazan. The formazan crystals were then dissolved in DMSO (100 μL) and the absorbance (450 nm) measured using a Thermomax Molecular Device microplate reader. The concentration capable of reducing cellular viability by 50% (CC_50_) was calculated, and the selectivity index (SI) determined using the following equation: A549 CC_50_/*C. haemulonii* complex GM-MIC.

### Effects of test compounds on biofilm-growing cells

The effect of the compounds on biofilm-growing cells was determined using the previously described microtiter-based technique (Ramage et al., 2001). The fungi (10^6^ yeast cells) were incubated for 48 h at 37°C in 96-well polystyrene microtiter plates (Corning®, Corning Incorporated, USA) containing Sabouraud-dextrose broth to accommodate biofilm formation. Following biofilm formation, the culture medium was aspirated and non-adherent cells removed by thoroughly washing the wells three times with sterile PBS (0.15 M NaCl, 0.01 M phosphate buffer, pH 7.2). Afterwards, the compounds, at the same concentrations used in the planktonic assays, were added to the wells, and the plates were incubated for further 48 h at 37°C. The metabolic activity was then evaluated using a colorimetric assay that measures the metabolic reduction of 2,3-bis (2-methoxy-4-nitro-5-sulfophenyl)-5-[(phenylamino) carbonyl]-2H-tetrazolium hydroxide (XTT; Sigma-Aldrich) to a water-soluble, brown formazan product (Peeters et al., 2008). The biofilm MIC (bMIC) for each compound was established as the minimal concentration capable of inhibiting 50% of metabolic activity when compared to compound-free wells (Ziccardi et al., 2015).

### Statistics

All the experiments were performed in triplicate and in three independent experimental sets. Statistical differences were analyzed by one-way ANOVA and Student's *t*-test, using GraphPad Prism version 5.0 software. In all analyses, *P* < 0.05 were considered statistically significant.

## Results

### Chelate synthesis

**Figure 1:**
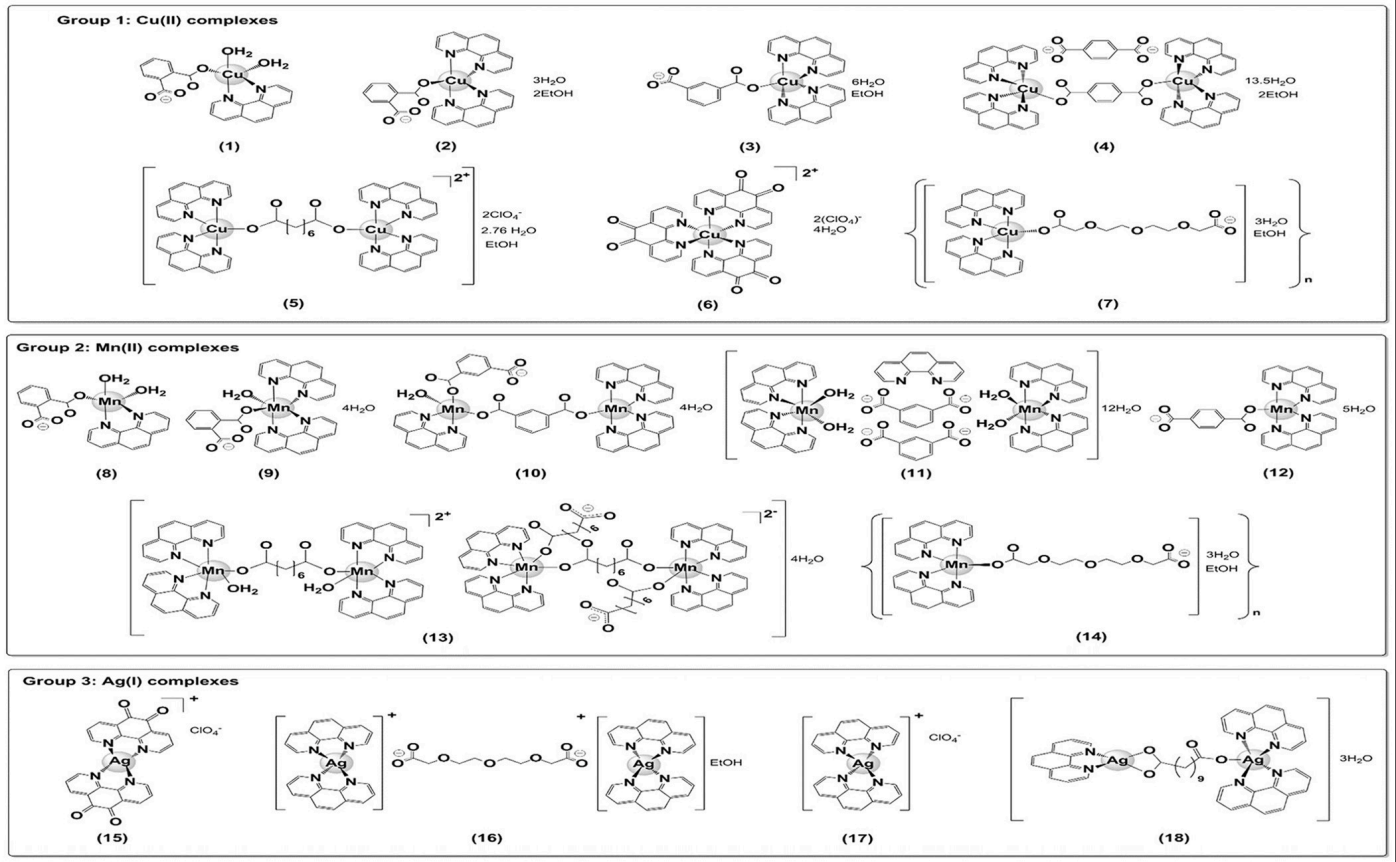
Structures of the copper(II) (1-7), manganese(II) (8-14), and silver(I) (15-18) chelates.

With the exceptions of {[Cu(3,6,9-tdda)(phen)_2_]3H_2_O.EtOH}_n_ (**7**) and [Ag_2_(3,6,9-tdda)(phen)_4_].EtOH (**16**), all of the other copper(II), manganese(II) and silver(I) chelates were prepared and characterized as previously described in the material and methods section. The formulation of chelate **7** was established using elemental analysis, IR spectroscopy and magnetic susceptibility measurements. Chelate **16** was characterized and formulated based on satisfactory elemental analyses and its IR and ^1^H-NMR spectra. The chemical structures of chelates **1–18** are presented in Figure 1 (in cases where exact solid state structures have not been established using X-ray crystallography tentative structures have been assigned).

### Effects of test compounds on planktonic fungal growth and their toxicity toward mammalian cells

The capacities of the metal chelates, **1–18**, to inhibit the viability of the nine clinical isolates belonging to the *C. haemulonii* complex are summarized in Table 1. The manganese(II) and silver(I) chelates significantly inhibited the viability of all the fungal isolates. With the exceptions of **1**, **2**, and **4**, the copper(II) complexes also demonstrated moderate to good antifungal activity. When the isolates where incubated in the presence of the simple salts CuCl_2_ and MnCl_2_, no appreciable effects against the fungal cells were observed. In contrast, the simple silver salt, AgNO_3_, and the metal-free phen and phendione ligands appreciably deterred fungal growth, although not as much as most of the manganese(II) and silver(I) chelates. Aqueous solutions of DMSO, when used at the concentrations used to make up solutions of the metal chelates, were inactive against all the fungal isolates (data not shown).

**Table 1:** Effect of test compounds on planktonic growth of the clinical isolates belonging to the *C. haemulonii* complex.

**Compounds**	***Candida haemulonii***				***Candida haemulonii*** **var**. ***vulnera***				***Candida duobushaemulonii***				
	**MIC (mg/L)**			**GM-MIC (mg/L)**	**MIC (mg/L)**			**GM-MIC (mg/L)**	**MIC (mg/L)**			**GM-MIC (mg/L)**	**Overall GM-MIC (mg/L)**
	**LIP *Ch*4**	**LIP *Ch*7**	**LIP *Ch*12**		**LIP *Ch*5**	**LIP *Ch*9**	**LIP *Ch*11**		**LIP *Ch*1**	**LIP *Ch*6**	**LIP *Ch*8**		
**Caspofungin**	0.5	0.25	0.125	0.25	0.25	0.25	0.5	0.315	0.125	0.25	0.125	0.157	0.23 (0.18 μM)
**1,10-phenanthroline** (phen)	1	1	1	1	1	1	0.5	0.79	1	1	1	1	0.93 (5.16 μM)
**1,10-phenanthroline-5,6-dione** (phendione)	2	2	2	2	2	2	2	2	2	4	2	2.52	2.16 (10.27 μM)
**CuCl**_2_	>32	>32	>32	>32	>32	>32	>32	>32	>32	>32	>32	>32	>32 (>23 μM)
**MnCl**_2_	>32	>32	>32	>32	>32	>32	>32	>32	>32	>32	>32	>32	>32 (>28 μM)
**AgNO**_3_	1	0.5	1	0.79	0.5	0.5	0.5	0.5	0.5	0.5	1	0.63	0.63 (3.93 μM)
**COPPER(ii) CHELATES**													
[Cu(ph)(phen)(H_2_O)_2_] (**1**)	>32	>32	>32	>32	>32	>32	>32	>32	>32	>32	>32	>32	>32 (>72 μM)
[Cu(ph)(phen)_2_].3H_2_O.2EtOH (**2**)	>32	>32	>32	>32	>32	>32	>32	>32	>32	>32	>32	>32	>32 (>43.52 μM)
[Cu(isoph)(phen)_2_].6H_2_O.EtOH (**3**)	8	8	16	10.08	4	8	8	6.35	4	8	4	5.04	6.86 (9.21 μM)
[{Cu(phen)_2_}_2_(terph)](terph).13.5H_2_O.2EtOH (**4**)	>32	>32	>32	>32	32	32	32	32	>32	>32	>32	>32	>32 (>21.92 μM)
[Cu_2_(oda)(phen)_4_](ClO_4_)_2_.2.76H_2_O.EtOH (**5**)	8	8	16	10.08	8	16	8	10.08	8	8	8	8	9.33 (7.09 μM)
[Cu(phendione)_3_](ClO_4_)_2_.4H_2_O (**6**)^*^	16	16	16	16	4	8	8	6.35	8	16	4	8	9.33 (9.65 μM)
{[Cu(3,6,9-tdda)(phen)_2_].3H_2_O.EtOH}_*n*_ (**7**)	4	4	2	3.17	2	2	4	2.52	2	2	2	2	2.52 (3.37 μM)
**MANGANESE(ii) CHELATES**													
[Mn(ph)(phen)(H_2_O)_2_] (**8**)	8	4	4	5.04	4	4	4	4	8	4	4	5.04	4.67 (10.71 μM)
[Mn(ph)(phen)_2_(H_2_O)].4H_2_O (**9**)	2	2	2	2	2	2	2	2	2	2	2	2	2 (2.98 μM)
[Mn_2_(isoph)_2_(phen)_3_].4H_2_O (**10**)	8	8	4	6.35	4	4	4	4	8	8	4	6.35	5.44 (5.15 μM)
{[Mn(phen)_2_(H_2_O)_2_]}_2_(isoph)_2_(phen).12H_2_O (**11**)	4	2	2	2.52	4	2	2	2.52	2	4	2	2.52	2.52 (1.54 μM)
[Mn(tereph)(phen)_2_].5H_2_O (**12**)	4	4	4	4	4	4	4	4	4	4	4	4	4 (5.96 μM)
[Mn_2_(oda)(phen)_4_(H_2_O)_2_][Mn_2_(oda)(phen)_4_(oda)_2_]4H_2_O (**13**)	2	2	2	2	2	2	2	2	2	4	2	2.52	2.16 (0.87 μM)
{[Mn(3,6,9-tdda)(phen)_2_].3H_2_O.EtOH}_*n*_ (**14**)	2	2	2	2	2	2	2	2	4	4	2	3.17	2.33 (3.15 μM)
**SILVER(i) CHELATES**													
[Ag(phendione)_2_]ClO_4_(**15**)^*^	1	1	1	1	2	1	1	1.26	2	2	2	2	1.36 (2.16 μM)
[Ag_2_(3,6,9-tdda)(phen)_4_].EtOH (**16**)	1	1	1	1	1	0.5	1	0.79	2	1	1	1.26	1 (0.83 μM)
[Ag(phen)_2_]ClO_4_ (**17**)^*^	1	1	1	1	1	0.5	0.5	0.63	2	1	2	1.59	1 (1.76 μM)
[Ag_2_(phen)_3_(udda)].3H_2_O (**18**)	0.125	0.25	0.125	0.16	0.5	0.125	0.25	0.25	0.5	0.25	1	0.5	0.27 (0.26 μM)

*All the MIC values are shown as mg/L. GM-MIC—Geometric mean of the compound MIC values (mg/L) for each species of Candida haemulonii complex. The last column presents the overall MIC geometric mean values for each of the test compounds across all nine strains of the C. haemulonii complex. The MIC values (μM) between copper(II), manganese(II) and silver(I) chelates were statistically significant (P = 0.038, one-way ANOVA)*.

* *Statistical analysis using one-way ANOVA evidenced that the MIC values of this compound among the three species were significant (compound **6**—P = 0.05; compound **15**—P = 0.02; compound **17**—P = 0.04) the uncoordinated dicarboxylic acid ligands (ie phthalic, isothphalic, terethphalicoctanedioic and 3,6,9-trioxaundecanedioic acids) do not exhibit anti-Candida capability (Devereux et al., 2000). The effect of classical antifungal agents against all these clinical strains of C. haemulonii species complex used herein were previously published by our research group (Ramos et al., 2015). In a general way, all the fungal strains were resistant to azoles (fluconazole, itraconazole and voriconazole) and amphotericin B; on the other hand, caspofungin was active against them. So, caspofungin was used as a positive control of classical antifungal agent able to inhibit the growth of all the clinical isolates used herein*.

From an analysis of the GM-MIC values (Table 1) calculated for each copper(II) chelate across all of the nine isolates of *C. haemulonii* complex, {[Cu(3,6,9-tdda)(phen)_2_]3H_2_O.EtOH}_n_ (**7**) showed the best anti-*Candida* activity (GM-MIC = 3.37 μM). [Mn_2_(oda)(phen)_4_(H_2_O)_2_][Mn_2_(oda)(phen)_4_(oda)_2_].4H_2_O (**13**) was the most active of the manganese(II) compounds (GM-MIC = 0.87 μM), and this was superseded by the silver(I) compounds, [Ag_2_(phen)_3_(udda)].3H_2_O (**18**) and [Ag_2_(3,6,9-tdda)(phen)_4_].EtOH (**16**) (GM-MIC = 0.26 and 0.83 μM, respectively). Indeed, all of the silver(I) chelates were more active than the simple silver(I) salt, AgNO_3_ (3.87 μM). In general, metal-free phen was ~2-fold more active than phendione.

The calculated GM-MIC values against each of the fungal species showed some degree of variation. For instance, the GM-MIC values of all the copper(II) compounds (excluding the ones with a GM-MIC value of 32 mg/L or higher) were lower against *C. duobushaemulonii* species when compared to the *C. haemulonii*. The same applies when comparing *C. duobushaemulonii* and *C. haemulonii* var. *vulnera*, except for the chelate **6**. Contrarily, the silver(I) compounds demonstrated an opposite trend, with *C. duobushaemulonii* having the higher GM-MIC values for all of the chelates when compared to both of the other *Candida* species.

The cytotoxicity of chelates **1–18**, the simple metal salts and the metal-free phen and phendione ligands, was assessed using the MTT assay with the lung epithelial cell lineage, A549 (Table 2). The manganese(II)-based complexes were extremely well tolerated by the A549 cells and, consequently, they exhibited the highest SI values (mean SI range >236.40–48.19) and with [Mn_2_(oda)(phen)_4_(H_2_O)_2_][Mn_2_(oda)(phen)_4_(oda)_2_].4H_2_O (**13**) featuring most prominently. Of the remaining test compounds, only water-soluble [Ag_2_(phen)_3_(udda)].3H_2_O (**18**) and AgNO_3_ returned relatively high SI values (mean SI = 56.11 and 27.43, respectively).

**Table 2:** Cytotoxicity and selectivity index values of the test compounds.

**Compounds**	**A549 (CC_50_)**	*C. haemulonii* (SI)^*^	***C. haemulonii* var. *vulnera* (SI)**	***C. duobushaemulonii* (SI)**	**Overall mean (SI)**
**1,10-phenanthroline** (phen)	4.30	4.30	5.42	4.30	4.67
**1,10-phenanthroline-5,6-dione** (phendione)	2.02	1.01	1.01	0.80	0.94
**CuCl_2_**	123.68	>3.87	>3.87	>3.87	>3.87
**MnCl_2_**	133.76	>4.18	>4.18	>4.18	>4.18
**AgNO_3_**	16.98	21.39	33.96	26.95	27.43
**COPPER(II) CHELATES**					
[Cu(ph)(phen)(H_2_O)_2_] (**1**)	0.53	<0.02	<0.02	<0.02	<0.02
[Cu(ph)(phen)_2_].3H_2_O.2EtOH (**2**)	1.93	<0.06	<0.06	<0.06	<0.06
[Cu(isoph)(phen)_2_].6H_2_O.EtOH (**3**)	0.89	0.09	0.14	0.18	0.14
[{Cu(phen)_2_}_2_(terph)](terph).13.5H_2_O.2EtOH (**4**)	3.86	<0.12	0.12	<0.12	ND
[Cu_2_(oda)(phen)_4_](ClO_4_)_2_.2.76H_2_O.EtOH (**5**)	0.98	0.10	0.10	0.12	0.11
[Cu(phendione)_3_](ClO_4_)_2_.4H_2_O (**6**)	0.68	0.04	0.11	0.09	0.08
{[Cu(3,6,9-tdda)(phen)_2_].3H_2_O.EtOH}_*n*_ (**7**)	1.06	0.33	0.42	0.53	0.43
**MANGANESE(II) CHELATES**					
[Mn(ph)(phen)(H_2_O)_2_] (**8**)	234.51	46.53	58.63	46.53	50.56
[Mn(ph)(phen)_2_(H_2_O)].4H_2_O (**9**)	259.34	129.67	129.67	129.67	129.67
[Mn_2_(isoph)_2_(phen)_3_].4H_2_O (**10**)	255.87	40.30	63.97	40.30	48.19
{[Mn(phen)_2_(H_2_O)_2_]}_2_(isoph)_2_(phen).12H_2_O (**11**)	251.76	99.91	99.91	99.91	99.91
[Mn(tereph)(phen)_2_].5H_2_O (**12**)	251.94	62.99	62.99	62.99	62.99
[Mn_2_(oda)(phen)_4_(H_2_O)_2_][Mn_2_(oda)(phen)_4_(oda)_2_]4H_2_O (**13**)	>512	>256	>256	>203.19	>236.40
{[Mn(3,6,9-tdda)(phen)_2_].3H_2_O.EtOH}_*n*_ (**14**)	261.67	130.84	130.84	82.42	114.70
**SILVER(I) CHELATES**					
[Ag(phendione)_2_]ClO_4_(**15**)	3.76	3.76	2.98	1.88	2.87
[Ag_2_(3,6,9-tdda)(phen)_4_].EtOH (**16**)	2.21	2.21	2.78	1.75	2.25
[Ag(phen)_2_]ClO_4_ (**17**)	2.07	2.07	3.29	1.30	2.22
[Ag_2_(phen)_3_(udda)].3H_2_O (**18**)	13.63	86.55	54.52	27.26	56.11

* *Selectivity indexes (SI) were calculated using the formula CC_50_/GM-MIC. Adenocarcinomic human alveolar basal epithelial A549 cells were used for the cytotoxicity assays and the CC_50_ values are expressed as mg/L. GM is the geometric mean of the SI values of the compounds against each species of the Candida haemulonii complex*.

### Effects of test compounds on biofilm-growing fungal cells

In this set of experiments, phen, phendione, AgNO_3_, and the metal-based chelates with overall GM-MIC values of 10 μM or less were selected in order to examine their capacity to disrupt fungal viability after biofilm formation on a polystyrene surface. The results (Table 3) revealed that the metal-based compounds were capable of interfering with the biofilm viability in a manner that was to some degree dependent upon the fungal isolate forming the *C. haemulonii* complex. Overall, the copper(II) chelate **7**, the manganese(II) compounds **11**, **13**, and **14** and the silver(I) compound **16** were the most active, presenting bMIC values below 10 μM. The remaining test complexes had bMIC values ranging from 12.04 to 28.42 μM. Metal-free phendione and AgNO_3_ were the least effective agents. Curiously, against several of the test compounds the *C. haemulonii* isolate, LIP*Ch*4, returned bMIC values exceeding the maximum concentration tested (512 mg/L).

**Table 3:** Inhibitory effects of test compounds on biofilm-growing cells of the clinical isolates of the *Candida haemulonii* complex.

**Compounds**	***Candida haemulonii***				***Candida haemulonii*** **var**. ***vulnera***			***Candida duobushaemulonii***					
	**bMIC (mg/L)**			**GM-bMIC (mg/L)**	**bMIC (mg/L)**			**GM-bMIC (mg/L)**	**bMIC (mg/L)**			**GM-bMIC (mg/L)**	**Overall GM-bMIC (mg/L)**
	**LIP *Ch*4**	**LIP *Ch*7**	**LIP *Ch*14**		**LIP *Ch*5**	**LIP *Ch*9**	**LIP *Ch*13**		**LIP *Ch*1**	**LIP *Ch*6**	**LIP *Ch*8**		
**1,10-phenanthroline** (phen)	512	2	2	12.70	4	32	8	10.08	1	1	1	1	5.04 (27.96 μM)
**1,10-phenanthroline-5,6-dione** (phendione)	>512	8	16	11.31^*^	16	16	32	20.16	4	32	16	12.70	14.67 (76 μM)^*^
**AgNO**_3_	>512	8	32	16^*^	128	64	8	40.32	16	16	128	32	29.34 (172 μM)^*^
**COPPER(II) CHELATES**													
[Cu(isoph)(phen)_2_].6H_2_O.EtOH (**3**)	32	16	16	20.16	16	8	16	12.70	32	8	8	12.70	14.81 (19.95 μM)
[Cu_2_(oda)(phen)_4_](ClO_4_)_2_.2.76H_2_O.EtOH (**5**)	32	16	16	20.16	32	8	16	16	32	8	16	16	17.28 (13.19 μM)
[Cu(phendione)_3_](ClO_4_)_2_.4H_2_O (**6**)^*^	256	32	16	50.80	32	32	32	32	16	16	8	12.70	27.43 (28.42 μM)
{[Cu(3.6.9-tdda)(phen)_2_].3H_2_O.EtOH}_*n*_ (**7**)	32	4	4	8	4	4	2	3.17	16	4	8	8	5.88 (7.9 μM)
**MANGANESE(II) CHELATES**													
[Mn(ph)(phen)(H_2_O)_2_] (**8**)	>512	4	16	8^*^	32	16	8	16	16	8	8	10.08	11.31 (25.98 μM)^*^
[Mn(ph)(phen)_2_(H_2_O)].4H_2_O (**9**)	512	2	16	25.40	16	4	4	6.35	16	4	2	5.04	9.33 (13.93 μM)
[Mn_2_(isoph)_2_(phen)_3_].4H_2_O (**10**)	>512	8	32	16^*^	32	8	8	12.70	16	16	16	16	14.67 (13.96 μM)^*^
{[Mn(phen)_2_(H_2_O)_2_]}_2_(isoph)_2_(phen).12H_2_O (**11**)	>512	8	32	16^*^	16	4	8	8	8	8	4	6.35	8.72 (5.3 μM)^*^
[Mn(tereph)(phen)_2_].5H_2_O (**12**)	>512	16	16	16^*^	2	4	16	5.04	32	8	16	16	10.37 (15.48 μM)^*^
[Mn_2_(oda)(phen)_4_(H_2_O)_2_][Mn_2_(oda)(phen)_4_(oda)_2_]4H_2_O (**13**)	>512	32	16	22.63^*^	16	4	8	8	4	4	8	5.04	8.72 (3.5 μM)^*^
{[Mn(3.6.9-tdda)(phen)_2_].3H_2_O.EtOH}_n_ (**14**)	512	4	4	20.16	16	4	8	8	16	4	16	10.08	11.76 (4.7 μM)
**SILVER(I) CHELATES**													
[Ag(phendione)_2_]ClO_4_(**15**)^*^	>512	8	16	11.31^*^	32	32	32	32	8	32	4	10.08	16 (25.48 μM)^*^
[Ag_2_(3.6.9-tdda)(phen)_4_].EtOH (**16**)	32	16	8	16	4	1	4	2.52	4	8	8	6.35	6.35 (5.2 μM)
[Ag(phen)_2_]ClO_4_ (**17**)^*^	32	32	8	20.16	8	0.5	8	8	16	8	8	10.08	12.34 (21.73 μM)
[Ag_2_(phen)_3_(udda)].3H_2_O (**18**)	32	8	16	16	8	0.5	16	11.31	16	8	8	10.08	12.34 (12.04 μM)

*All biofilm MIC (bMIC) values are shown in mg/L. The values of bMIC were statistically different between Candida haemulonii strains and the other two species (P = 0.04. t-test). No significant differences were detected when comparing C. haemulonii var. vulnera and C. duobushaemulonii. The values of bMIC were statistically different between the strains (P ≤ 0.0001. one-way ANOVA)*.

* *These values do not include the results obtained with the LIP Ch4 strain that could not be established (>512)*.

## Discussion

Invasive opportunistic mycoses are associated with high morbidity and mortality rates, representing serious health challenges and creating a significant burden for both patients and health care systems due to the elevated treatment costs (Caggiano et al., 2015). The reduced number of available effective antifungal drugs and the increasing emergence of resistance profiles have indubitably limited the treatment options of such infections (Abu-Elteen and Hamad, 2012; Almeida et al., 2012; Muro et al., 2012; Ramos et al., 2015; Sanguinetti et al., 2015). The severe difficulties encountered in the treatment of infections caused by the *C. haemulonii* complex obviates the need to explore alternative therapeutic approaches.

The antimicrobial capabilities of metals have been harnessed for centuries, with historical applications in water and food preservation, agriculture and medicine (Lemire et al., 2013). Inorganic medicinal chemistry, an interdisciplinary research field, is advancing our knowledge of metal toxicity and facilitating the design of metal-containing compounds as effective and targeted antimicrobials, offering a realistic alternative to organic antibiotics (Lemire et al., 2013). We have previously demonstrated that Cu(II), Mn(II), and Ag(I) chelates containing phen-type ligands exhibit antifungal activity against clinical isolates of *C. albicans* (Devereux et al., 2000; Geraghty et al., 2000; Coyle et al., 2003; McCann et al., 2004; Creaven et al., 2009). Therefore, we were prompted to assess the antifungal chemotherapeutic potential of this class of metal chelate against the highly resistant *C. haemulonii* species complex. To this end, we tested 16 known chelates (**1–6**, **8–15**, **17**, and **18**) and 2 new ones (**7** and **16**), containing either neutral phen or phendione ligands and coordinated or uncoordinated dicarboxylate or perchlorate anions (Figure 1). The complexes, along with the “metal-free” phen and phendione and simple metal salts, were assessed for their ability to inhibit the growth of nine clinical strains of the three species that make up the *C. haemulonii* complex against both planktonic and biofilm lifestyles. Potent antifungal activity was observed for a number of the test compounds, with the order of planktonic growth inhibition being; chelate **18**>**16**>**13**>**17**>**11**>**15**>**9**>**14**>**7**>**10**>**12**>**5**>**3**>**6**>**8**. When the chelates are classified according to the nature of the central metal ion, the silver(I) compounds (**15–18**) have the best overall growth inhibitory effect, followed by those of manganese(II) (**8–14**).

While the simple metal salts, CuCl_2_ and MnCl_2_, were not able to effectively reduce the fungal cell proliferation, the silver(I) salt exhibited good inhibitory capability. It should be noted that AgNO_3_ is a well-established source of Ag^+^ ions, which are well known to have potent antifungal activity (Lemire et al., 2013). Thus, it is fair to assume that the type of metal ion present in the compounds may directly impact their antifungal capability (Devereux et al., 2000; McCann et al., 2004). In contrast, the uncoordinated dicarboxylic acid ligands (e.g., phthalic, isothphalic, terethphalicoctanedioic, and 3,6,9-trioxaundecanedioic acids) are known to not exhibit anti-*Candida* capability, and our results indicate that these ligands also have no effect on the proliferation of the fungal isolates employed in the current study (data not shown) (Devereux et al., 2000). The metal-free (uncoordinated) phen and phendione also had a potent inhibitory effect on the *C. haemulonii* species complex, which is in agreement with results obtained from other studies reporting the activity of phen against *Candida* species such as *C. tropicalis, C. krusei* and *C. glabrata* (Geraghty et al., 2000; McCann et al., 2000, 2004, 2012; Coyle et al., 2003). It should be noted that, although metal-free phen and phendione and the Ag^+^ ions (from AgNO_3_) exhibited antifungal activities, the silver(I) chelates (**15–18**) were able to induce a more pronounced inhibitory antifungal effect based on their overall GM-MIC (μM values). The same observation applies to the copper(II), **7**, and the manganese(II), **9**, **10, 11**, **13**, and **14**, chelates, which were more active in inhibiting cellular proliferation than both the corresponding simple metal salt and the “metal-free” phen and phendione ligands, indicating that the chelates are much superior antimicrobial agents. Previous studies into the mode of action of these chelates revealed their potential to disrupt mitochondrial activity and respiration processes, restriction of cell growth by interfering with protein synthesis, cleavage of proteins, DNA interaction/cleavage and epigenetic influences (Devereux et al., 2000; Metcalfe and Thomas, 2003; McCann et al., 2012; Kharissova et al., 2014). These mechanisms of action of the metal chelates differ from those of the azole and polyene drug classes commonly used to treat fungal infections, and they offer the prospect of developing inorganic drugs capable of overcoming the resistance traits that make the treatment of *C. haemulonii* species complex infection so difficult.

An ability to form biofilms is an acknowledged virulence factor of microorganisms, which protects them from the host's immune system and also from the action of antifungal drugs (Mello et al., 2017). Administered drugs experience reduced access to the pathogen as a result of the presence of a biofilm matrix composed of polysaccharides, carbohydrates, proteins and DNA. Besides that, the presence of a biofilm on an implanted medical device may become a reservoir of pathogenic cells that are released to the bloodstream, resulting in dissemination to internal sites and organs (Bujdáková, 2016; Ramos et al., 2017). *Candida* strains with an inherent ability to establish a biofilm are associated with a higher mortality rate when compared to strains incapable of biofilm formation (Peeters et al., 2008; Garcia-Vidal et al., 2013; Kawai et al., 2015; Rajendran et al., 2016). The *C. haemulonii* complex has a considerable biofilm-forming capacity (Bujdáková, 2016; Ramos et al., 2017) and, curiously, the presence of a central venous catheter was mentioned in all of the fungemia case reports regarding these species between 2002 and 2010 (Khan et al., 2007; Almeida et al., 2012; Cendejas-Bueno et al., 2012; Muro et al., 2012). One documented case also describes an outbreak in neonatal patients who were receiving total parenteral nutrition (Kim et al., 2009).

Metal-free phen and phendione, AgNO_3_ and the chelates **3** and **5–18**, which all had shown good *in vitro* activity against planktonic growth of the three fungal species forming the *C. haemulonii* complex, were also assessed for their capacity to inhibit the growth of cells in a mature biofilm. The most active were the manganese(II) chelates **13** (3.5 μM), **14** (4.7 μM) and the silver(I) complex **16** (5.2 μM). Two more compounds were also very effective against the biofilm, the manganese(II) complex **11** and the copper(II) complex **7**. A clear reduction in the antifungal capacity of the test compounds was clearly evident on going from planktonic to biofilm cells. The most pronounced decrease in activity was detected for [Ag_2_(phen)_3_(udda)].3H_2_O (**18**) (46.3-fold), followed by AgNO_3_ (43.8-fold), **17** (12.3-fold), **15** (11.8-fold) and complex **16** (6.3-fold). It is interesting that chelate **18**, which was the most active against planktonic cell growth, showed the highest reduction in activity. A degree of variation in compound tolerance amongst the fungal constituent strains of the biofilm is noticeable, with *C. haemulonii* LIP*Ch*4 being the most defiant. The data obtained in the current study demonstrates that although the test compounds were less active against a biofilm, some had impressively low overall GM-bMIC values, such as chelates **13** (3.5 μM), **14** (4.7 μM), **16** (5.2 μM), and **11** (5.3 μM).

In a global scale, there is an urgent demand for the discovery of new therapies with effective, safe and cheap drugs to be used as alternative treatment against (multi)drug-resistant pathogens. In this line of thinking, copper(II), manganese(II) and silver(I) chelates containing phen and phendione ligands are relatively cheap and easy to prepare and they clearly have significant antifungal chemotherapeutic potential against the highly resistant *C. haemulonii* complex.

## Conclusions

In summary, the results from the present study indicate that copper(II), manganese(II) and silver(I) chelates containing phen and phendione ligands are capable of inhibiting planktonic and biofilm growth of the three fungal species that comprise the *C. haemulonii* complex, and which are known to be highly resistant to the most commonly used antifungals. In particular, a number of the manganese(II) and silver(I) complexes were very effective at preventing cellular proliferation and the manganese(II) complexes demonstrated relatively low cytotoxicity toward the mammalian cell line, A549, indicating very favorable selectivity for the *C. haemulonii* species complex.

## Author contributions

RG, MB, and AS conceived and designed the study. RG, PM, MF, LR, TM, and AA performed the experiments. All the authors analyzed the data. MM, MD, MB, and AS contributed reagents, materials, and/or analysis tools. RG, MM, MD, MB, and AS wrote and revised the paper. All the authors contributed to the research and approved the final version of the manuscript. All the authors agree to be accountable for all aspects of the work.

### Conflict of interest statement

The authors declare that the research was conducted in the absence of any commercial or financial relationships that could be construed as a potential conflict of interest.

## References

[B1] Abu-Elteen K. H. Hamad M. A. (2012). Changing epidemiology of classical and emerging human fungal infections: a review. Jordan J. Biol. Sci. 5, 215–230.

[B2] Almeida J. N. Jr. Motta A. L. Rossi F. Abdala E. Pierrotti L. C. Kono A. S. G.. (2012). First report of a clinical isolate of *Candida haemulonii* in Brazil. Clinics 67, 1229–1231. 10.6061/clinics/2012(10)18 23070353 PMC3460029

[B3] Bujdáková H. (2016). Management of *Candida* biofilms: state of knowledge and new options for prevention and eradication. Future Microbiol. 11, 235–251. 10.2217/fmb.15.139 26849383

[B4] Caggiano G. Coretti C. Bartolomeo N. Lovero G. De Giglio O. Montagna M. T. (2015). *Candida* bloodstream infection in Italy: changing epidemiology during 16 years of surveillance. Biomed. Res. Int. 2015, 256580. 10.1155/2015/256580 26064890 PMC4439500

[B5] Casey M. T. McCann M. Devereux M. Curran M. Cardin C. Convery M. (1994). Synthesis and structure of the Mn_2_ (II,II) complex salt [Mn_2_(oda)(phen)_4_(H_2_O)_2_] [Mn_2_(oda)_2_(phen)_4_] (odaH_2_ = octanedioic acid): a catalyst for H_2_O_2_ disproportionation. J. Chem. Soc. Chem. Commun. 22, 2643–2645. 10.1039/C39940002643

[B6] Cendejas-Bueno E. Kolecka A. Alastruey-Izquierdo A. Theelen B. Groenewald M. Kostrzewa M.. (2012). Reclassification of the *Candida haemulonii* complex as *Candida haemulonii* (*C. haemulonii* group I), *C. duobushaemulonii* sp. nov. (*C. haemulonii* group II), and *C. haemulonii* var. *vulnera* var. nov.: three multiresistant human pathogenic yeasts. J. Clin. Microbiol. 50, 3641–3651. 10.1128/JCM.02248-12 22952266 PMC3486233

[B7] CLSI (2008). Clinical and Laboratory Standards Institute. Reference Method for Broth Dilution Antifungal Susceptibility Testing of Yeasts: Approved Standard –3rd Edn M27-A3. Wayne, PA: CLSI.

[B8] Coyle B. Kavanagh K. McCann M. Devereux M. Geraghty M. (2003). Mode of anti-fungal activity of 1,10-phenanthroline and its Cu(II), Mn(II) and Ag(I) complexes. Biometals 16, 321–329. 10.1023/A:1020695923788 12572690

[B9] Creaven B. S. Devereux M. Karcz D. Kellett A. McCann M. Noble A.. (2009). Copper(II) complexes of coumarin-derived schiff bases and their anti-*Candida* activity. J. Inorg. Biochem. 103, 1196–1203. 10.1016/j.jinorgbio.2009.05.017 19631386

[B10] Creaven B. S. Egan D. A. Karcz D. Kavanagh K. McCann M. Mahon M.. (2007). Synthesis, characterisation and antimicrobial activity of copper(II) and manganese(II) complexes of coumarin-6,7-dioxyacetic acid (cdoaH2) and 4-methylcoumarin-6,7-dioxyacetic acid (4-MecdoaH2): X-ray crystal structures of [Cu(cdoa)(phen)_2_].8.8H_2_O and [Cu(4-Mecdoa)(phen)_2_].13H_2_O (phen=1,10-phenanthroline). J. Inorg. Biochem. 101, 1108–1119. 10.1016/j.jinorgbio.2007.04.010 17555821

[B11] Devereux M. McCann M. Cronin J. F. Ferguson G. McKee V. (1999). Binuclear and polymeric copper(II) dicarboxylate complexes: synthesis and crystal structures of [Cu_2_(pda)(phen)_4_](ClO_4_)_2_.5H_2_O.EtOH, [Cu_2_(oda)(phen)_4_](ClO_4_)_2_.2.67H_2_O.EtOH and {Cu_2_(pda)_2_(NH_3_)_4_(H_2_O)_2_.4H_2_O}_*n*_ (odaH_2_ = octanedioic acid; pdaH_2_ = pentanedioic acid; phen = 1,10-phenanthroline). Polyhedron 18, 2141–2148. 10.1016/S0277-5387(99)00100-X

[B12] Devereux M. McCann M. Leon V. Geraghty M. McKee V. Wikaira J. (2000). Synthesis and biological activity of manganese (II) complexes of phthalic and isophthalic acid: X-ray crystal structures of [Mn(ph)(Phen)_2_(H_2_O)].4H_2_O, [Mn(Phen)_2_(H_2_O)_2_]_2_ (Isoph)_2_ (Phen)·14H_2_O and {[Mn(Isoph)(bipy)_2_]_4_.2.75bipy}_n_ (phH_2_ = phthalic acid; Isoph = isophthalic acid; Phen = 1,10-phenanthroline; bipy = 2,2-bipyridine). Met. Based Drugs 7, 275–288. 10.1155/MBD.2000.275 18475957 PMC2365232

[B13] Garcia-Vidal C. Viasus D. Carratalá J. (2013). Pathogenesis of invasive fungal infections. Curr. Opin. Infect. Dis. 26, 270–276. 10.1097/QCO.0b013e32835fb920 23449139

[B14] Geraghty M. Cronin J. F. Devereux M. McCann M. (2000). Synthesis and antimicrobial activity of copper(II) and manganese(II) α,ω-dicarboxylate complexes. Biometals 13, 1–8. 10.1023/A:1009271221684 10831218

[B15] Giusiano G. Mangiaterra M. Saito V. G. Rojas F. Gómez V. Díaz M. C. (2005). Etiology of fungaemia and catheter colonization in Argentinian paediatric patients. Mycoses 49, 49–54. 10.1111/j.1439-0507.2005.01184.x 16367819

[B16] Kawai A. Yamagishi Y. Mikamo H. (2015). *In vitro* efficacy of liposomal amphotericin B, micafungin and fluconazole against non-*albicans* species biofilms. J. Infect. Chemother. 21, 647–653. 10.1016/j.jiac.2015.05.007 26141813

[B17] Kellett A. Howe O. O'Connor M. McCann M. Creaven B. S. McClean S.. (2012). Radical induced DNA damage by cytotoxic square-planar copper(II) complexes incorporating *o*-phthalate and 1,10-phenanthroline or 2,2′-dipyridyl. Free Radic. Biol. Med. 53, 564–576. 10.1016/j.freeradbiomed.2012.05.034 22659117

[B18] Kellett A. O'Connor M. McCann M. McNamara M. Lynch P. Rosair G.. (2011). Bis-phenanthroline copper(II) phthalate complexes are potent *in vitro* antitumour agents with ‘self-activating’ metallo-nuclease and DNA binding properties. Dalton Trans. 40, 1024–1027. 10.1039/c0dt01607a 21165464

[B19] Khan Z. U. Al-Sweih N. A. Ahmad S. Al-Kazemi N. Khan S. Joseph L.. (2007). Outbreak of fungemia among neonates caused by *Candida haemulonii* resistant to amphotericin B, itraconazole and fluconazole. J. Clin. Microbiol. 45, 2025–2027. 10.1128/JCM.00222-07 17428940 PMC1933024

[B20] Kharissova O. V. Mendez-Rojas M. A. Kharisov B. I. Méndez U. O. Martínez P. E. (2014). Metal complexes containing natural and artificial radioactive elements and their applications. Molecules 19, 10755–10802. 10.3390/molecules190810755 25061724 PMC6272025

[B21] Kim M. N. Shin J. H. Sung H. Lee K. Kim E. C. Ryoo N.. (2009). *Candida haemulonii* and closely related species at 5 university hospitals in Korea: identification, antifungal susceptibility, and clinical features. Clin. Infect. Dis. 48, 57–61. 10.1086/597108 19193113

[B22] Lemire J. A. Harrison J. J. Turner R. J. (2013). Antimicrobial activity of metals: mechanisms, molecular targets and applications. Nat. Rev. Microbiol. 11, 371–384. 10.1038/nrmicro3028 23669886

[B23] Leon V. (2000). Synthesis, Characterization and Catalytic and Biological Activity of New Manganese(II) Carboxylate Complexes [dissertation]. Dublin Institute of Technology, Dublin.

[B24] McCann M. Coyle B. McKay S. McCormack P. Kavanagh K. Devereux M.. (2004). Synthesis and X-ray crystal structure of [Ag(phendio)_2_]ClO_4_ (phendio = 1,10-phenanthroline-5,6-dione) and its effects on fungal and mammalian cells. Biometals 17, 635–645. 10.1007/s10534-004-1229-5 15689107

[B25] McCann M. Geraghty M. Devereux M. O'Shea D. Mason J. O'Sullivan L. (2000). Insights into the mode of action of the anti-*Candida* activity of 1,10-phenanthroline and its metal chelates. Met. Based Drugs 7, 185–193. 10.1155/MBD.2000.185 18475944 PMC2365221

[B26] McCann M. Kellett A. Kavanagh K. Devereux M. Santos A. L. S. (2012). Deciphering the antimicrobial activity of phenanthroline chelators. Curr. Med. Chem. 19, 2703–2714. 10.2174/092986712800609733 22455581

[B27] McCann S. McCann M. Casey M. T. Devereux M. McKee V. McMichael P. (1997). Manganese(II) complexes of 3,6,9-trioxaundecanedioic acid (3,6,9-tddaH_2_): X-ray crystal structures of [Mn(3,6,9-tdda)(H_2_O)_2_].2H_2_O and {[Mn(3,6,9-tdda)(phen)2].3H2O.EtOH}n. Polyhedron 16, 4247–4252. 10.1016/S0277-5387(97)00233-7

[B28] Mello T. P. Ramos L. S. Braga-Silva L. A. Branquinha M. H. Santos A. L. S. (2017). Fungal biofilm – a real obstacle against an efficient therapy: lessons from *Candida*. Curr. Top. Med. Chem. 17, 1987–2004. 10.2174/1568026617666170105145227 28056742

[B29] Metcalfe C. Thomas A. (2003). Kinetically inert transition metal complexes that reversibly bind to DNA. Chem. Soc. Rev. 32, 215–224. 10.1039/b201945k 12875027

[B30] Mosmann T. (1983). Rapid colorimetric assay for cellular growth and survival: application to proliferation and cytotoxicity assays. J. Immunol. Methods 65, 55–63. 6606682 10.1016/0022-1759(83)90303-4

[B31] Muro M. D. Motta F. A. Burger M. Melo A. S. A. Dalla-Costa L. M. (2012). Echinocandin resistance in two *Candida haemulonii* isolates from pediatric patients. J. Clin. Microbiol. 50, 3783–3785. 10.1128/JCM.01136-12 22895037 PMC3486200

[B32] Nett J. E. (2014). Future directions for anti-biofilm therapeutics targeting *Candida*. Expert. Revi. Anti. Infect. Ther. 12, 375–382. 10.1586/14787210.2014.885838 24506174

[B33] Peeters E. Nelis H. J. Coenye T. (2008). Comparison of multiple methods for quantification of microbial biofilms grown in microtiter plates. J. Microbiol. Methods 72, 157–165. 10.1016/j.mimet.2007.11.010 18155789

[B34] Rajendran R. Sherry L. Nile C. J. Sherriff A. Johnson E. M. Hanson M. F.. (2016). Biofilm formation is a risk factor for mortality in patients with *Candida albicans* bloodstream infections-Scotland, 2012-2013. Clin. Microbiol. Infect. 22, 87–93. 10.1016/j.cmi.2015.09.018 26432192 PMC4721535

[B35] Ramage G. Robertson S. N. Williams C. (2014). Strength in numbers: antifungal strategies against fungal biofilms. Int. J. Antimicrob. Agents 43, 114–120. 10.1016/j.ijantimicag.2013.10.023 24359842

[B36] Ramage G. Walle K. V. Wickes B. L. López-Ribot J. L. (2001). Standardized method for *in vitro* antifungal susceptibility testing of *Candida albicans* biofilms. Antimicrob. Agents Chemother. 45, 2475–2479. 10.1128/AAC.45.9.2475-2479.2001 11502517 PMC90680

[B37] Ramos L. S. Figueireido-Carvalho M. H. Barbedo L. S. Ziccardi M. Chaves A. L. Zancopé-Oliveira R. M.. (2015). *Candida haemulonii* complex: species identification and antifungal susceptibility profiles of clinical isolates from Brazil. J. Antimicrob. Chemother. 70, 111–115. 10.1093/jac/dku321 25134720

[B38] Ramos L. S. Oliveira S. C. S. Branquinha M. H. Santos A. L. S. (2017). Planktonic growth and biofilm formation profiles in *Candida haemulonii* species complex. Med. Mycol. 10.1093/mmy/myx005 28159990

[B39] Richardson M. Lass-Flörl C. (2008). Changing epidemiology of systemic fungal infections. Clin. Microbiol. Infec. 4, 5–24. 10.1111/j.1469-0691.2008.01978.x 18430126

[B40] Ruan S. Y. Kuo Y. W. Huang C. T. Hsiue H. C. Hsueh P. R. (2010). Infections due to *Candida haemulonii*: species identification, antifungal susceptibility and outcomes. Int. J. Antimicrob. Agents 35, 85–88. 10.1016/j.ijantimicag.2009.08.009 19786341

[B41] Sanguinetti M. Posteraro B. Lass-Flörl C. (2015). Antifungal drug resistance among *Candida* species: mechanisms and clinical impact. Mycoses 58, 2–13. 10.1111/myc.12330 26033251

[B42] Thornton L. Dixit V. Assad L. O. Ribeiro T. P. Queiroz D. D. Kellett A.. (2016). Water-soluble and photo-stable silver(I) dicarboxylate complexes containing 1,10-phenanthroline ligands: antimicrobial and anticancer chemotherapeutic potential, DNA interactions and antioxidant activity. J. Inorg. Biochem. 159, 120–132. 10.1016/j.jinorgbio.2016.02.024 26986979

[B43] Ziccardi M. Souza L. O. P. Gandra R. M. Galdino A. C. Baptista A. R. Nunes A. P.. (2015). *Candida parapsilosis* (*sensu lato*) isolated from hospitals located in the Southeast of Brazil: species distribution, antifungal susceptibility and virulence attributes. Int. J. Med. Microbiol. 305, 848–859. 10.1016/j.ijmm.2015.08.003 26319940

